# A cholesterol‐coupled *N*‐acetyl‐aspartyl‐glutamate metabolic network facilitates the neuroprotective impact of progesterone‐estradiol crosstalk in neurons

**DOI:** 10.1002/alz70855_100111

**Published:** 2025-12-23

**Authors:** Kim Hei‐Man Chow

**Affiliations:** ^1^ School of Life Sciences, The Chinese University of Hong Kong, Shatin, Hong Kong, Hong Kong

## Abstract

**Background:**

Sex differences have been demonstrated in AD. The exact mechanism of sex biases in brain diseases remains unclear but likely involves an interplay between the endocrine and the nervous systems. Estrogen and progesterone, the primary ovarian hormones dominant at different cycle stages, can exert opposing effects to the brain. Randomized clinical trials and observational studies also revealed that the peri‐menopausal phase of the life cycle of aging women is particularly associated with declines in memory function, increased risks of mild cognitive impairment and dementia. Despite so, the potential connection between hormone replacement therapy use and the risk of dementia remains a subject of ongoing debate for years, and that the intrinsic cellular changes underlying the enhanced disease vulnerability observed in postmenopausal women remain elusive.

**Method:**

Mining of brain transcriptomic and metabolomic datasets from the ROSMAP cohort was performed. *In vivo* behavioral analyses, brain transcriptomic, metabolomic and electrophysiological analyses were conducted in an accelerated ovarian failure mouse model, ERRα knockout, and the Lafala‐AD transgenic mouse models.

**Result:**

Elevated estradiol to progesterone ratio (i.e., estrogen dominance) during the onset of perimenopause is correlated with elevated risk to cognitive and memory decline. Human brain transcriptomic and metabolomic changes pointed to the impaired function of ERRα (estrogen‐related receptor alpha). Progesterone‐guided estrogen receptor signaling stimulates ERRα activity indirectly by its actions on neuronal cholesterol homeostasis. Consequently, this prevents truncation of the TCA cycle at succinate dehydrogenase, which would otherwise cause a net catabolic shift of *N*‐acetyl‐aspartyl‐glutamate (NAAG) driven by an adaptive aspartate‐dependent response that attempts to reconstruct a “mini‐cycle”. The free glutamate released alongside the net catabolism of NAAG is stochastically released pre‐synaptically, thereby increasing spontaneous neuronal activities. Coupled with the bioenergetic incompetency that occurs during estrogen dominance, this slowly depletes cellular ATP and increases susceptibility to energy crises triggered by additional excitatory insults, such as age‐related intraneuronal accumulation of APP or Aß.

**Conclusion:**

Collectively, our work reveals an unforeseen interaction cholesterol balance and mitochondrial energy production in neurons. The findings also shed light on the potential impact of estrogen dominance during the perimenopausal transition, contributing to increased susceptibility to AD/ADRD among aging women.

